# Molecular Profiling of Well-Differentiated Neuroendocrine Tumours: The Role of ctDNA in Real-World Practice

**DOI:** 10.3390/cancers14041017

**Published:** 2022-02-17

**Authors:** Angela Lamarca, Melissa Frizziero, Jorge Barriuso, Zainul Kapacee, Wasat Mansoor, Mairéad G. McNamara, Richard A. Hubner, Juan W. Valle

**Affiliations:** 1 Department of Medical Oncology, The Christie NHS Foundation Trust, Manchester M20 4BX, UK; melissa.frizziero@cruk.manchester.ac.uk (M.F.); jorge.barriuso@manchester.ac.uk (J.B.); zainul.kapacee@nhs.net (Z.K.); was.mansoor@nhs.net (W.M.); mairead.mcnamara@nhs.net (M.G.M.); richard.hubner@nhs.net (R.A.H.); juan.valle@nhs.net (J.W.V.); 2 Division of Cancer Sciences, Faculty of Biology, Medicine and Health, University of Manchester, Manchester M13 9PL, UK

**Keywords:** neuroendocrine, molecular profiling, targeted therapies, mutation, fusion, ctDNA

## Abstract

**Simple Summary:**

The analysis of circulating tumour DNA (ctDNA) can help to identify genetic alterations present in cancer cells without the need to access tumour tissue, which can be an invasive approach. This study explored the feasibility of analysing ctDNA in patients with advanced well-differentiated neuroendocrine tumours (WdNETs). A total of 45 patients (15 with WdNETs) were included. Although feasible (with a non-evaluable sample rate of 27.8%), mutation-based ctDNA analysis was of limited clinical utility for patients with advanced WdNETs. While patients with WdNETs could still be offered genomic profiling (if available and reimbursed), it is important to manage patients’ expectations regarding the likelihood of the results impacting their treatment.

**Abstract:**

*Background*: The role of tumour genomic profiling in the clinical management of well-differentiated neuroendocrine tumours (WdNETs) is unclear. Circulating tumour DNA (ctDNA) may be a useful surrogate for tumour tissue when the latter is insufficient for analysis. *Methods*: Patients diagnosed with WdNETs underwent ctDNA genomic profiling (FoundationLiquid^®^); non-WdNETs (paraganglioma, goblet cell or poorly-differentiated neuroendocrine carcinoma) were used for comparison. The aim was to determine the rate of: test failure (primary end-point), “pathological alterations” (PAs) (secondary end-point) and patients for whom ctDNA analysis impacted management (secondary end-point). *Results*: Forty-five patients were included. A total of 15 patients with WdNETs (18 ctDNA samples) were eligible: 8 females (53.3%), median age 63.2 years (range 23.5–86.8). Primary: small bowel (8; 53.3%), pancreas (5; 33.3%), gastric (1; 6.7%) and unknown primary (1; 6.7%); grade (G)1 (*n* = 5; 33.3%), G2 (9; 60.0%) and G3 (1; 6.7%); median Ki-67: 5% (range 1–30). A total of 30 patients with non-WdNETs (34 ctDNA samples) were included. Five WdNETs samples (27.78%) failed analysis (vs. 17.65% in non-WdNETs; *p*-value 0.395). Of the 13 WdNET samples with successful ctDNA analyses, PAs were detected in 6 (46.15%) (vs. 82.14% in non-WdNETs; *p*-value 0.018). In WdNETs, the PA rate was independent of concomitant administration anti-cancer systemic therapies (2/7; 28.57% vs. 4/6; 66.67%; *p*-value 0.286) at the time of the ctDNA analysis: four, one and one samples had one, two and three PAs, respectively. These were: *CDKN2A* mutation (mut) (one sample), *CHEK2*mut (one), *TP53*mut (one), *FGFR2* amplification (one), *IDH2*mut (one), *CTNNB1*mut (one), *NF1*mut (one) and *PALB2*mut (one). None were targetable (0%) or impacted clinical management (0%). There was a lower maximum mutant allele frequency (mMAF) in WdNETs (mean 0.33) vs. non-WdNETs (mean 26.99), even though differences did not reach statistical significance (*p*-value 0.0584). *Conclusions:* Although feasible, mutation-based ctDNA analysis was of limited clinical utility for patients with advanced WdNETs. The rates of PAs and mMAFs were higher in non-WdNETs. While patients with WdNETs could still be offered genomic profiling (if available and reimbursed), it is important to manage patients’ expectations regarding the likelihood of the results impacting their treatment.

## 1. Introduction

Neuroendocrine neoplasms (NENs) are broadly classified according to their morphological differentiation and proliferative rate in well-differentiated neuroendocrine tumours (WdNETs) (grade (G)1–2 (Ki-67 < 20%) or G3 (Ki-67 ≥ 20%, usually ≤50%)) and in poorly differentiated neuroendocrine carcinomas (PdNECs) (always G3, Ki-67 ≥ 20%) [1]. However, this histopathological classification only partially captures the biological heterogeneity within this family of tumours, and a more granular biological subtyping is needed to deliver more personalised treatment to patients with NENs.

The molecular profiling of tumours is becoming of increasing relevance in the management of patients with advanced cancer due to its potential to identify targetable molecular alterations and predictive biomarkers that can inform new treatments. In relation to the use of next-generation sequencing (NGS) technologies in neuroendocrine neoplasms (NENs), the current recommendation from the European Society for Medical Oncology (ESMO) is to assess the tumour mutational burden (TMB), an estimate of the rate of somatic mutations within a tumour genome, in WdNETs [2], as this may predict the tumour’s response to immunotherapy. This recommendation is based on the results of a prospective exploratory analysis of the multi-cohort phase II KEYNOTE-158 trial, which assessed the activity of the programmed death-1 inhibitor Pembrolizumab in previously treated patients with 10 different cancer types, including NETs. This analysis reported a response rate of 29% in patients with a high TMB (≥10 mutations/megabase (Mb)) using targeted NGS in diagnostic tumour tissues, as opposed to 6% in patients with a lower TMB [3].

Overall, the published literature supports the use of the multi-omic profiling of NENs as a tool to better understand their underlying biology and to identify the NEN molecular subtypes with potential clinical implications [4]. One of the largest studies in this regard was published by Scarpa and colleagues, who explored the whole-genome landscape of 102 sporadic pancreatic NETs (PanNETs) [5]. This study showed that 17% of PanNETs harbour germline mutations affecting DNA repair genes (e.g., *MUTYH*, *CHEK2* and *BRCA2*), or the genes *MEN1* and *VHL*. Somatic mutations or fusions are most commonly found in genes involved in four pathways: chromatin remodelling, DNA damage repair, mTOR signalling activation and telomere maintenance. Integrative transcriptomic analysis identified an additional PanNET subgroup associated with hypoxia and HIF signalling.

Van Riet and colleagues explored the genomic landscape of 85 advanced NENs (69 WdNETs and 16 PdNECs) of different primary origin: 68 from different gastro-entero-pancreatic (GEP) sites, 7 from the lung and 12 of unknown origin [6]. They showed a relatively high average TMB of 5.45 somatic mutations/Mb, with *TP53, KRAS, RB1, CSMD3, APC, CSMD1, LRATD2, TRRAP* and *MYC* as major drivers in PdNECs, compared to an overall low TMB in WdNETs (average of 1.09 somatic mutations/Mb), with the different repertoires of gene drivers affected by somatic aberrations in pancreatic (*MEN1, ATRX, DAXX, DMD* and *CREBBP*) and midgut (*CDKN1B*) NETs. 

Hong et al. assessed the mutational and copy number variation (CNV) profiles of 211 PanNETs, confirming that insulinomas had different genomic features than other non-functional (NF)-PanNETs [7], and reclassified these tumours into novel molecular subtypes. Some of the subgroups identified were associated with a higher relapse risk.

The newly defined G3-WdNETs [8] have also been genomically characterised. Williamson and colleagues showed that G3-WdNETs of pancreatic origin exhibited a *TSC1*-disrupting fusion and a *CHD7–BEND2* fusion, and lacked any somatic variants in *ATRX, DAXX* and *MEN1* [9]. 

There are two main challenges to incorporating the molecular profiling of NENs into standard clinical practice. Firstly, the clinical utility of molecular profiling beyond the determination of TMB remains unclear, especially from a therapeutic perspective [2]. In relation to the targetable alterations identified, 42 of 85 samples (49%) from the patients with advanced NEN, in a series explored by Van Riet and colleagues, harboured a potential therapeutic target, with a predominance of NEC within these patients (15/42; 36%), followed by PanNETs (11/42; 26%) [6]. These targetable alterations were associated with the available “on-label” treatment options in 21 cases; in the other 21, they were associated with “off-label” therapies. Secondly, adequate profiling requires a minimum of 20% of tumour content; this might be difficult to achieve as the NEN tumour tissue remaining after a standard histopathological diagnostic work-up is usually of poor quantity or quality, and the efficient recovery of DNA/RNA from archival tumour tissues is challenging. In addition, it is extremely difficult to make a decision about the right technology to apply (whole-genome, whole-exome or RNA-sequencing) in an extremely volatile context regarding the cost and constant evolution of technology. Cell-free DNA may offer an easily accessible, alternative source of fresh tumour material for genomic characterisation; the profiling of its DNA fraction, namely ctDNA, has proven informative and clinically useful in different cancer types, and may also find application in patients with NENs [10,11]. In addition, ctDNA readouts, if detectable, can be measured over time to monitor changes in tumour burden and genomic profile.

This study aimed to assess the feasibility of ctDNA molecular profiling using a targeted NGS platform in patients with WdNETs, and its potential to guide clinical management.

## 2. Methods

Patients previously diagnosed with advanced NENs underwent molecular profiling (ctDNA) using the FoundationLiquid^®^ testing platform (72 cancer-related genes) between April and November 2019, in the framework of a collaboration between The Christie NHS Foundation Trust (Manchester, UK) and Foundation Medicine (Roche^®^, Basel, Switzerland). This platform allows for the identification of pathogenic and likely pathogenic somatic and germline variants, herein defined as “pathological alterations,” including base substitutions, insertions, deletions, copy number alterations and chromosomal rearrangements. It also reports on high microsatellite instability (MSI-h). Patients provided written informed consent for molecular profiling to be performed; in addition, the retrospective analysis of these data was approved by the institutional Audit Committee (approval number 19/2634).

Patients with a histologically confirmed WdNET diagnosis, as per the 2019 World Health Organisation Classification parameters (WHO editorial Board, 2019), were included in this analysis; patients diagnosed with non-WdNETs, such as paraganglioma, goblet cell adenocarcinoma or PdNECs, were used for comparative purposes only. Clinical baseline characteristics, demographic and treatment data were collected. Molecular profiling information was extracted, including the success of sample analysis, the presence or absence of pathological alterations and the mutant allele frequency (MAF) for pathological alterations. 

The aim of the study was to assess the feasibility and the clinical impact of ctDNA molecular profiling in WdNETs. The primary end-point was to assess the percentage of WdNET ctDNA samples that failed testing (defined as those scenarios where insufficient DNA was isolated for analysis). Secondary end-points included defining the proportion of the sample in which pathological findings were identified, and the percentage of patients for whom management changed based on molecular profiling results. 

Descriptive statistical analysis using STATA v.12 was performed. The Chi-Square test, Fisher’s exact test and *t*-test were used, when appropriate. A two-sided *p*-value < 0.05 was considered statistically significant.

## 3. Results

Samples from 45 patients were included: 15 WdNETs and 30 non-WdNETs. 

### 3.1. Patient Characteristics

Within the total of the 15 individual patients with WdNETs (accounting for 18 ctDNA samples) (Table 1), 8 were female (53.33%), with a median age of 63.2 years (range 23.5–86.8). Most were small-bowel-primary patients (8 patients; 53.33%) (pancreas (5; 33.33%), gastric (1; 6.67%) and unknown primary (1; 6.67%)) and grade 2 patients (9; 60.00%) (grade 1 (5; 33.33%), grade 3 (1; 6.67%)), with a median Ki-67 of 5% (range 1–30). All patients with WdNET had a metastatic disease and seven were on treatment (three somatostatin analogues; four chemotherapy) at the time of the ctDNA sample acquisition. 

### 3.2. Feasibility and Main Findings of ctDNA-Based Molecular Profiling 

**Figure 1:**
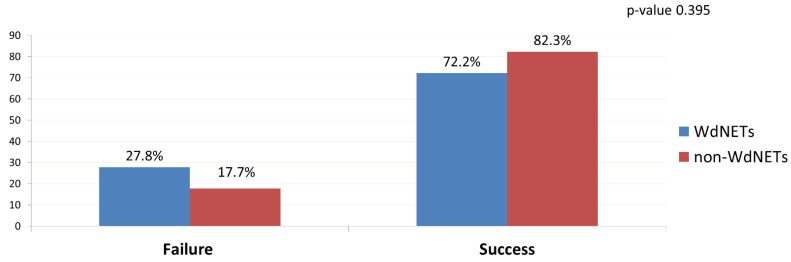
Failure and success rate of molecular profiling analysis by population being analysed.

A total of 5 WdNETs samples (27.78%) failed analysis (vs. 17.65% in non-WdNETs; *p*-value 0.395) (Figure 1). 

**Figure 2:**
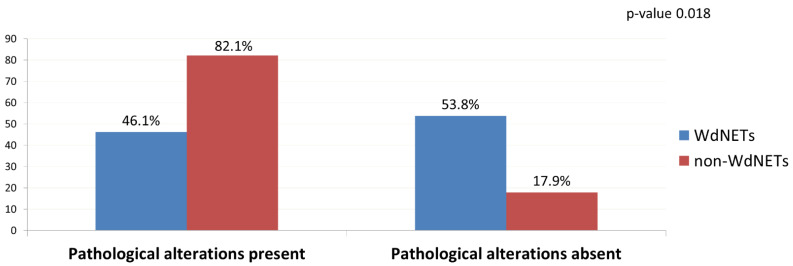
Presence and absence of pathological alterations in ctDNA by population being analysed.

**Figure 3:**
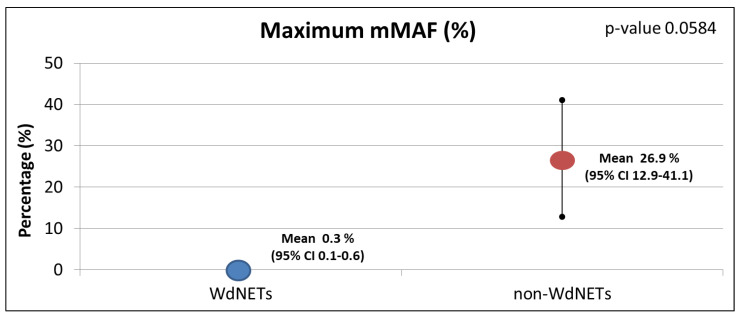
Maximum mutant allele frequency (mMAF) by population analysed.

Of the 13 WdNET samples with a successful ctDNA analysis, pathological alterations were identified in 6 (46.15%) (vs. 82.14% in non-WdNETs; *p*-value 0.018) (Figure 2). In addition, there was a lower maximum MAF in WdNETs (mean 0.33) vs. non-WdNETs (mean 26.99), even though differences did not reach statistical significance (*p*-value 0.0584) (Figure 3). The rate of findings of unclear significance was similar between WdNETs (69.23%) and non-WdNETs (78.57%) (*p*-value 0.517).

**Figure 4:**
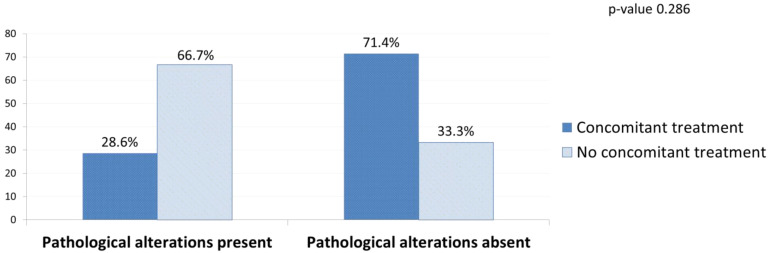
Presence and absence of pathological alterations in ctDNA by concomitant systemic treatment (WdNETs cohort).

Within the WdNET cohort, there was a higher presence of pathological mutations in G2 tumours (grade 1: 0%, grade 2: 66.67%; *p*-value 0.07) and in patients who were not receiving ongoing, concomitant anti-cancer systemic therapy at the time of the ctDNA sampling (no treatment: 66.67%, on treatment: 28.57%; *p*-value 0.286; Figure 4).

### 3.3. Identified Pathological Alterations and Impact on Management

A total of six pathological alterations were identified within the WdNET samples, including the *CDKN2A* mutation (one sample), *CHEK2* mutation (one sample), *TP53* mutation (two samples), *FGFR2* amplification (one sample), *IDH2* mutation (one sample), *CTNNB1* mutation (one sample), *NF1* mutation (one sample) and *PALB2* mutation (one sample). Concomitant alterations were identified in two samples (one had two alterations (the *CHEK2* and *TP53* mutations) and another had three (the *CTNNB1, NF1* and *PALB2* mutations)). The other four samples had one unique pathological alteration each. 

None (0% of samples) of the identified pathological findings were considered potentially targetable. Thus, ctDNA-based molecular profiling did not change therapeutic management for any of the patients with a WdNET (0% of patients). 

## 4. Discussion

Although feasible, the role of molecular profiling using ctDNA seems to be limited in clinical decision making for patients with advanced WdNETs. The rate of identification of pathological alterations and the reported mMAF were significantly lower than in non-WdNETs. This is despite WdNETs having a similar sample failure rate to that described for non-Wd NETs, suggesting that the results are not associated with an increased rate of analysis failure in WdNETs (or a small amount of tumour-derived DNA in the bloodstream), but rather with a lower prevalence of significant alterations in this group of patients. Therefore, molecular profiling may be more relevant in non-WdNETs than in WdNETs. This is in line with the findings of genomic profiling studies of NEN tumour tissue showing that somatic mutations are less frequent in WdNETs than in PdNECs [6,12]. 

Although molecular profiling of WdNETs has been widely utilised to better understand the biology of these malignancies, true precision medicine therapeutic approaches in this patient group are currently non-existent [13]. Other series exploring targetable alterations in WdNETs have reported a higher rate of targetable alterations [6,10], which may be due to the differing definitions of “targetable” used, which ideally should follow evidence-based definitions [14]. It would also be of interest to understand how many of those patients were actually matched to a specific treatment based on the molecular alteration identified. While there are widely available data on this for other malignancies, data in WdNETs are scarce. The MOSCATO-01 clinical trial prospectively evaluated the clinical benefit of utilising high-throughput genomic analyses to identify actionable molecular alterations and match patients with a specific targeted therapy [15]. Of the total of 1035 patients included, 199 patients were matched with a specific treatment; within the group that received matched treatment, 11% of patients achieved an objective response, and a progression-free survival (PFS)2/PFS1 ratio of >1.3 was identified in 33% of patients. Ten patients with “thyroid and other endocrine glands” were included in this study. Of these, only two received a “matched” treatment. This corroborates the challenges of identifying targetable alterations in NENs.

Despite in-depth research on the identification of relevant molecular pathways in NETs [16], the development of precision medicine approaches represents one of the most relevant challenges in the current management of patients with NENs [17]. Beyond developments in the arena of nuclear medicine, which is rapidly developing new theragnostic approaches [18], predictive biomarkers for systemic therapy selection in WdNETs are lacking [19,20,21].

Interestingly, the findings of this study corroborate previous evidence suggesting that the dysregulation of cell-cycle/DNA damage repair (e.g., *TP53, CDKN2A, CHEK2, PALB2*) is a recurrent, critical biological vulnerability of WdNETs [5], highlighting the rationale for its therapeutic exploitation. Ongoing trials are evaluating the potential role of targeting such alterations. In addition, trials evaluating peptide-receptor radionuclide therapies in combination with DNA damage repair inhibitors, for patients with WdNETs expressing somatostatin receptors (e.g., ClinicalTrials.gov NCT04086485, NCT05053854), do also exist.

In addition to the potential therapeutic impact of molecular alterations, the identification of specific, presumed somatic mutations in ctDNA should trigger germ-line testing in selected cases, where there is the potential for a known underlying hereditary syndrome in patients with WdNETs, such as multiple endocrine neoplasia (MEN) syndromes, Von Hippel–Lindau disease (VHL), Neurofibromatosis 1 (NF1) syndrome and Tuberous sclerosis (TS) [22].

The limitations of this study include the small sample size, the heterogeneity of the tumour type and treatment administered and the potential selection bias at the time of selecting patients for molecular profiling, as non-consecutive patients were considered for this. In addition, the series included a mix of advanced-stage and prior-line therapy patients, and there was no access to concomitant tissue profiling, which would have been of interest. However, a strength of this study was that all patients were tested with identical technologies and within the same time frame. In addition, the presence of a cohort of patients with non-WdNETs allowed us to put the findings into context, providing our results with more robustness and allowing for clinical interpretation. 

Finally, the NGS platform used here included 70 ‘pan-cancer’-related genes, yet excluded a number of genes commonly altered in WdNETs, such as *MUTYH, ATRX, DAXX* and *MEN1*; a WdNET-specific gene panel, developed on the basis of more recent NGS data from large NEN datasets, may allow for the increased sensitivity of ctDNA detection in these patients.

## 5. Conclusions

The use of molecular profiling utilising ctDNA in WdNETs is feasible, but the results are currently unlikely to identify targetable alterations that may impact patient management. While patients with WdNETs should still be offered molecular profiling (if available and reimbursed), it is important to manage patient expectations in relation to the likelihood of the results impacting their management. It is possible that, due to the nature of these malignancies, which have generally low numbers of somatic mutations, the evolution of the field from exclusive ctDNA profiling to a combination of mutational analyses and epigenetic changes (including methylation analyses) will have an impact on the expansion of molecular profiling’s use as a tool for neuroendocrine tumours, expanding from prognosis to the uncovering of new targets.

## Figures and Tables

**Table 1:** Baseline characteristics for patients who underwent ctDNA-based molecular profiling (WdNETs).

Baseline Characteristics (WdNETs)		Patients with Advanced WdNETs (*n* = 15)	
		*n*	%
Age (time of sample taken)	Median (range)	63.2	23.5–86.8
Gender	Female	8	53.3
	Male	7	46.7
Site of primary	Small bowel	8	53.3
	Pancreas	5	33.3
	Gastric	1	6.7
	Unknown primary	1	6.7
Grade	Grade 1	5	33.3
	Grade 2	9	60.0
	Grade 3	1	6.7
Ki-67	Median (range)	5	1–30
Concomitant treatment at time of ctDNA sampling	Yes	7	46.7

## Data Availability

Data can be made available upon request.
